# A Comparison of Anti-Nuclear Antibody Quantification Using Automated Enzyme Immunoassays and Immunofluorescence Assays

**DOI:** 10.1155/2014/534759

**Published:** 2014-01-28

**Authors:** Renata Baronaite, Merete Engelhart, Troels Mørk Hansen, Gorm Thamsborg, Hanne Slott Jensen, Steen Stender, Pal Bela Szecsi

**Affiliations:** ^1^Department of Clinical Biochemistry, Gentofte Hospital, University of Copenhagen, 2900 Hellerup, Denmark; ^2^Department of Rheumatology, Gentofte Hospital, University of Copenhagen, 2900 Hellerup, Denmark; ^3^Department of Rheumatology, Glostrup Hospital, University of Copenhagen, 2600 Glostrup, Denmark

## Abstract

Anti-nuclear antibodies (ANA) have traditionally been evaluated using indirect fluorescence assays (IFA) with HEp-2 cells. Quantitative immunoassays (EIA) have replaced the use of HEp-2 cells in some laboratories. Here, we evaluated ANA in 400 consecutive and unselected routinely referred patients using IFA and automated EIA techniques. The IFA results generated by two independent laboratories were compared with the EIA results from antibodies against double-stranded DNA (dsDNA), from ANA screening, and from tests of the seven included subantigens. The final IFA and EIA results for 386 unique patients were compared. The majority of the results were the same between the two methods (*n* = 325, 84%); however, 8% (*n* = 30) yielded equivocal results (equivocal-negative and equivocal-positive) and 8% (*n* = 31) yielded divergent results (positive-negative). The results showed fairly good agreement, with Cohen's kappa value of 0.30 (95% confidence interval (CI) = 0.14–0.46), which decreased to 0.23 (95% CI = 0.06–0.40) when the results for dsDNA were omitted. The EIA method was less reliable for assessing nuclear and speckled reactivity patterns, whereas the IFA method presented difficulties detecting dsDNA and Ro activity. The automated EIA method was performed in a similar way to the conventional IFA method using HEp-2 cells; thus, automated EIA may be used as a screening test.

## 1. Introduction

Visual inspection via indirect immunofluorescence microscopy has been the gold standard for detecting anti-nuclear antibodies (ANA) since their discovery more than 50 years ago [1, 2], and this method continues to be performed virtually with no modifications. Rodent tissue (stomach, liver, and/or kidney) was used as a substrate in early ANA testing but was subsequently replaced by the human epithelial-like cell line HEp-2 (HEp-2). Some laboratories have replaced HEp-2 cells with commercial cells (HEp-2000 cells) that overexpress Sjögren's Syndrome A antigen/small ribonucleoprotein particle (SSA/Ro) because HEp-2 cells lack sensitivity for the detection of the SSA/Ro antigens [3]. The intensity and staining patterns of antibodies that bind to cellular components allow a skilled observer to distinguish between numerous nuclear staining patterns: homogeneous, speckled, nuclear membranous, centromeric, nuclear dot, pleomorphic, SSA/Ro-positive, and other mixed or atypical patterns [4]. The IFA method is influenced by cell type, fixation procedure, dilution of patient serum, inspection time, day-to-day performance, experience level of the microscopist, and the microscope itself [5, 6]. Although the presence of ANA is associated with various rheumatic and nonrheumatic diseases, its highest sensitivity lies in identifying cases of systemic lupus erythematosus (SLE) (93–95%), systemic sclerosis (85%), juvenile idiopathic arthritis (61–80%), Sjögren's syndrome (48%), and mixed connective tissue disease (MCTD). ANA is also apparent in other autoimmune diseases and various nonrheumatologic conditions [4, 7–10]. Furthermore, a low ANA titer (1 : 40) is present in up to 32% of healthy individuals, whereas 3–5% exhibit a higher titer (1 : 160–1 : 320) [7]. The commercial availability of standardized kits has made IFA tests superior compared with homemade preparations. However, such kits remain at best only semiquantitative and cumbersome to perform despite attempts to automate IFA techniques [6, 8]. Several ANA antigens have been identified, and quantitative enzyme immunoassays (EIA) have been developed using either purified extracts or recombinant antigens. Most of the early manual EIA techniques have been widely replaced by automated versions. These newer methods are amenable to modern laboratories with high-throughput platforms, and they provide quantitative, reproducible results with minimal hands-on time and require less operator skill. In contrast to classical IFA methods using HEp-2 cells, which contain several hundred different antigens, the reactivity of EIA methods is limited to the relatively few individual antigens included in the assays. Although this factor may reduce EIA reactivity to some relevant antigens, it could also diminish reactivity toward irrelevant antigens.

Much of our experience with the clinical utility of ANA is based on standard IFA methodology. Thus, some reservations exist among clinicians as to whether EIA methods can replace conventional HEp-2 IFA techniques [1, 11, 12]. In this study, we compared conventional IFA with automated EIA for evaluating blood samples from 400 consecutive patients who were referred for routine ANA testing.

## 2. Materials and Methods

### 2.1. Patients

Between August and November 2007, serum samples were collected from 400 consecutive patients who were referred for routine ANA testing from hospital wards and outpatient clinics at three hospitals in the Copenhagen suburbs (Gentofte, Herlev, and Glostrup Hospitals). Information on the patients' age, sex, diagnosis, disease status, and any medications was collected one year after the ANA testing (registered anonymously). The study was conducted in accordance with the principles of the Declaration of Helsinki, but it was not considered a bioethics project according to the definition of the Danish Act on the Bioethics Committee System and the Processing of Bioethics Projects. Thus, no application for a review was submitted, and written informed consent was not obtained. The Danish Bioethics Committees for the Capital Region have approved the classification of this study.

### 2.2. Assays

**Figure 1:**
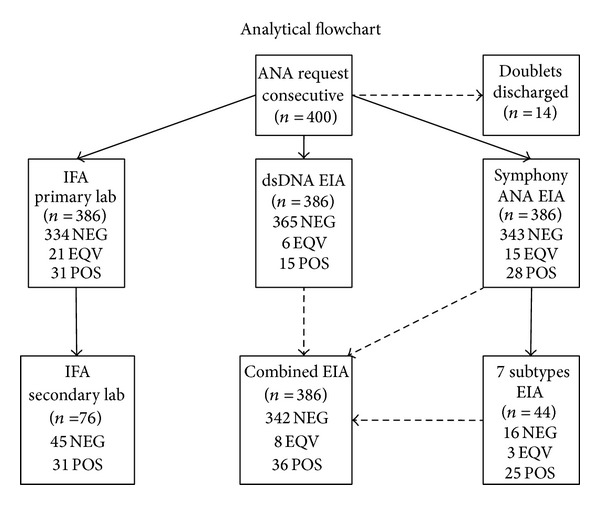
Analytical flowchart. EIA with a combination of dsDNA, ANA Symphony, and the 7 subantigens (SmD, U1RNP, SSA/Ro, SSB/La, Scl-70, CENP-B, and Jo-1) is referred to as “Combined EIA.” IFA tests were performed at a primary laboratory (Gentofte Hospital) and a secondary laboratory (Statens Serum Institut). NEG: negative, EQV: equivocal, POS: positive, and ND: not determined.

An analytical flowchart for this study is shown in Figure 1. IFA was performed by incubating a 1 : 160 dilution of serum with HEp-2000 cells, which overexpress the 60-kDa SSA/Ro antigen [3], according to the manufacturer's protocol (Immuno Concepts, Sacramento, CA, USA). In cases of positive or ambiguous results from the primary laboratory, the samples were reanalyzed and titrated blindly at a secondary laboratory (Statens Serum Institut, Copenhagen, Denmark) using the same IFA assay. Automated EIA for ANA (Symphony, a mixture of the following seven subantigens: purified recombinant SmD; SSA/Ro (52- and 60-kDa); SSB/La; Scl-70; CENP-B; U1RNP (RNP70, A, C); and Jo-1 proteins) and anti-dsDNA measurements were performed using EliA reagents and a UniCAP 100 instrument (Phadia, Freiburg, Germany). All antibody levels were classified according to the manufacturer's recommendations: anti-dsDNA < 10 IU/mL was considered negative, >15 IU/mL was positive, and 10–15 IU/mL was equivocal; and an ANA Symphony test sample to calibrator ratio < 0.7 was negative, >1.0 was positive, and = 0.7–1.0 was equivocal. Samples with a positive or equivocal Symphony result were reflex-tested by analyzing their individual reactivity to each of the seven subantigens included in the screening test. CENP-B, Jo-1, SSA/Ro, SSB/La, and Scl-70 levels < 7.0 U/mL and U1RNP and SmD levels < 5.0 U/mL were considered to be negative. CENP-B, Jo-1, SSA/Ro, SSB/La, and Scl-70 levels between 7.0 and 10.0 U/mL and U1RNP and SmD levels between 5.0 and 10.0 U/mL were considered equivocal. The final, combined EIA results (negative, equivocal, and positive) were derived from the data from the anti-dsDNA, ANA Symphony, and seven individual subantigen tests. The IFA results (negative, equivocal, and positive) were based on the data obtained from both the primary and the secondary laboratories. For the 14 patient serum samples that were tested in duplicate, only the results obtained from the first replicate were used.

### 2.3. Statistics

The VassarStats calculator (Vassar College, Poughkeepsie, NY, USA) was used to calculate the agreement between tests using Cohen's unweighted kappa with confidence intervals. A Kappa statistic < 0.2 was considered to indicate a “poor” strength of agreement; 0.21–0.40 was “fair,” 0.41–0.60 was “moderate,” 0.61–0.80 was “good,” and >0.8 was “very good” [13]. All the other data analyses were performed using SPSS, release 20.0 (IBM, Armonk, NY, USA).

## 3. Results

The study population consisted of 241 females with a mean age of 52.4 years (range: 3–92) and 145 males with a mean age of 52.2 years (range: 1–89). The diagnoses are listed in Table 1.

The median anti-dsDNA level was 1.7 IU/mL, with a 90% interpercentile range of 0.6–11.1. A total of 365 samples (94.6%) were found to be anti-dsDNA-negative, six (1.6%) were equivocal, and 15 (3.9%) were positive. The female patients exhibited a statistically nonsignificant trend toward higher anti-dsDNA values (mean: 5.3 versus 2.4 IU/mL). Four of the 28 Symphony-positive serum samples were also positive for anti-dsDNA.

The primary laboratory generally reported weak, homogenous results that were found to be negative in the secondary laboratory (Table 2). Nonetheless, the 76 samples analyzed via IFA at both the primary and secondary laboratories exhibited moderately good agreement, with Cohen's unweighted kappa value of 0.45 (95% CI = 0.28–0.62).

The final assessments of the 386 samples obtained via EIA (the combined anti-dsDNA, Symphony, and seven subantigen results) and via IFA (the combined results from the primary and secondary laboratories) were compared (Table 3). The majority of the results were in agreement (*n* = 325, 84%), whereas 8% (*n* = 30) yielded equivocal results (equivocal-negative and equivocal-positive) and 8% (*n* = 31) yielded divergent results (positive-negative) (Table 4). The results exhibited fairly good agreement, with a Cohen's kappa value of 0.30 (95% CI = 0.14–0.46). The kappa value decreased to 0.23 (95% CI = 0.06–0.40) when the results of the anti-dsDNA analyses were omitted from the calculation despite the few solitary reactions for dsDNA.

Of the 333 samples that were negative using IFA, 24 were found to be reactive with EIA (anti-dsDNA and/or Symphony) without a clear pattern. However, the IFA testing missed several positive samples with significant anti-dsDNA reactivity via EIA screening. Furthermore, the IFA exhibited decreased reliability for detecting Ro reactivity, either alone or in combination with the other antigens. In contrast, the IFA method detected 14 positive and 19 equivocal samples that were negative when screened using the EIA method. Although most of these IFA reactions were weak, six of the samples had clear nuclear reactivity and four had clear speckled reactivity.

The IFA and EIA results for patients with SLE or MCTD exhibited fair agreement, and the results for patients with scleroderma demonstrated good agreement. The IFA produced equivocal results for rheumatoid arthritis (RA) patients, primarily due to weak, homogeneous IFA reactions at the primary laboratory and negative reactions in both EIA and IFA methods at the secondary laboratory. The sera from an unexpectedly large proportion (3/12) of patients with osteoporosis reacted positively in EIA and/or IFA, and two of these cases were Ro-positive.

Of the 388 samples evaluated, 36 were positive for ANA using EIA, and 11 of these were solely reactive to anti-dsDNA. A total of 30 samples reacted positively using IFA, and 15 of these also reacted positively in the EIA tests. Only one of the samples that reacted positively using both IFA and EIA exhibited anti-dsDNA reactivity, and the degree of reactivity was borderline. Among the patients diagnosed with ANA-associated disease, 23% (16/70) had positive EIA results (two samples had equivocal results) and 24% (17/70) had positive IFA results (two samples had equivocal results). Seven patients with nonANA-associated rheumatic disease reacted positively in both EIA and IFA, and 12 samples showed equivocal results via IFA compared with only two via EIA. Both the EIA and IFA methods yielded positive results in four of the eight patients with SLE (in three cases, both tests were positive). The EIA-negative and IFA-positive samples exhibited a nuclear staining pattern via IFA at a titer of 1 : 160, and the IFA-negative and EIA-positive samples had relatively high reactivity to both dsDNA (38 IU/mL) and Ro (38.2 U/mL). Both assays yielded negative results in three cases, one of which represented an overlapping syndrome with MCTD. Of the three patients with undifferentiated connective tissue disease (UCTD), all reacted positively via IFA (high-titer speckled patterns in two cases and a high-titer homogeneous pattern in one case), whereas only one case reacted positively in the EIA tests (U1RNP and SmD). Of the remaining two cases, only one exhibited weak anti-dsDNA reactivity. Out of eight patients with juvenile RA, only one had a positive IFA result (a speckled pattern at a titer > 1,280), whereas that patient's EIA result was negative. Of the 48 adult cases of RA, four and three reacted positively via EIA and IFA, respectively. Both EIA and IFA yielded positive results in all three MCTD patients. All of these patients had high anti-U1RNP titers; one reacted positively to Ro, and one exhibited positive anti-SmD and anti-dsDNA reactivity. In these cases, the IFA pattern was observed at a high titer (>1 : 1,280) and was speckled or homogenous. Both the IFA and EIA results were negative for the single dermatomyositis patient and the two polymyositis patients.

Among the 316 patients without ANA-associated disease, 33 exhibited positive ANA reactivity (IFA, EIA, or both) with no obvious pattern. Among the 56 RA patients, four tested positive via EIA and four via IFA, with a single overlap. However, nine RA patients received weak, equivocal results via IFA.

There were unexpectedly large proportions of ANA-positive results among the patients with osteoporosis (3/12), vitamin D deficiency (3/5), and optic neuritis (3/13). Optic neuritis patients tested positive for anti-dsDNA only via EIA, whereas vitamin D-deficient patients showed reactivity to Ro and CENP-B via EIA and strongly speckled reactivity via IFA. The osteoporotic patients showed anti-dsDNA and anti-Ro reactivity via EIA; however, their IFA results included predominantly speckled and homogenous patterns.

## 4. Discussion

Our results demonstrate that at best using HEp-2000 cells as a substrate for IFA ANA testing only improves upon the insufficient sensitivity of SSA/Ro antibodies; this method still fails to detect even the extremely high antibody levels that are detected using the specific Ro EIA (Table 3). Both the screening and the solitary SSA/Ro EIA methods employ a mixture of the 52- and 60-kDa Ro protein isoforms; thus, they cannot identify reactivity toward a single Ro isoform, which might be clinically useful. Our data also illustrate the difficulties in reproducing IFA results, which have been previously observed by others [14]. The analyses of 76 samples at both the primary and secondary laboratories produced only a moderately good agreement (Table 2). Notably, both laboratories used identical commercial assay systems; all the factors except the microscopist were eliminated. An even lower degree of agreement could be expected among laboratories that use different methods [15].

However, there were weaknesses in this study that should be addressed. First, IFA was not performed at the secondary laboratory in all cases. Most of the samples with negative results at the primary laboratory were not validated further, which may have resulted in an under- or overestimation of the degree of agreement. However, because only one of the 23 negative samples analyzed at the primary laboratory was found to be positive at the second laboratory, the overall conclusion is likely to stand. Second, relatively few patients with ANA-associated disease (*n* = 44) were evaluated; thus, predictive values cannot be calculated for individuals with this disease.

Taken together, our results are consistent with previous reports of similar findings using EIA [16–18]. Fenger et al. compared the results for three selected populations evaluated using IFA and seven different EIA methods [18], but they evaluated anti-dsDNA and Symphony reactivity separately. Notably, the authors did not test for the individual antigens when the screening test revealed positive or equivocal results. However, they found a degree of agreement between EIA and IFA tests that was comparable to our results, although the Phadia tests exhibited similar specificity but lower sensitivity compared with the other assays.

Other groups have produced results similar to ours when comparing a combination of Phadia Symphony EIA screening and anti-dsDNA testing with IFA; however, their performance when testing sera from SLE patients was relatively lower [17]. We did not observe significant differences among the relatively few SLE cases in our study, although neither EIA nor IFA identified all the relevant patients. However, other groups have found that IFA and EIA exhibited satisfactory specificity and sensitivity for assessing SLE patients, albeit with somewhat variable levels of agreement [19–22].

Bizzaro et al. compared the findings of 16 manufacturers and two university laboratories, which used different methods to analyze sera from 11 autoimmune patients [21]. The overall agreement, independent of method, was relatively good for ANA (95.5%) and was somewhat lower for anti-dsDNA (85.2%). However, considerable variation between the different methods was observed for both IFA and EIA. The IFA results revealed variability in both titer and pattern, and the EIA results showed variability in specificity for individual antigens. In all cases, the EIA results were at a low, borderline cut-off level. In agreement with these observations, a multicenter evaluation of nine EIA kits could not clearly demonstrate that one assay was superior to the others [23], particularly for anti-dsDNA and SmD antigen detection, although the newer versions yielded improved performance.

HEp-2 cells contain several hundred antigens; therefore, IFA should be the ultimate multiplexing screening assay and should be principally similar to the Phadia Symphony and other EIA kits. However, some of the antigens in HEp-2 cells are not relevant to autoimmune diseases, whereas other more relevant antigens are present in only minor amounts.

Although IFA can compensate for some of these limitations via pattern recognition and titer determination, EIA can only reveal reactivity toward the limited number of antigens included in the test by using a more standardized quantitative method.

Phadia has introduced a new screening assay that evaluates 17 antigens (the EliA CTD Screen), but some authors still consider the sensitivity of this assay to be insufficient, especially for assessing anti-fibrillin and anti-RNA polymerase III reactivity [20]. Op de Beeck et al. recently compared the CTD assay against an IFA method with a HEp-2000 substrate, using samples from autoimmune and chronic fatigue patients, blood donors, and disease control patients [24]. They found that the CTD assay yielded high specificity but with limited sensitivity. Furthermore, an excessive number of samples required additional testing with all the individual antigens. These shortcomings could be attributed to the inclusion of a dsDNA antigen in the CTD screening assay and to an overabundance of conjugated antigens on a single surface, resulting in dilution of specific, individual signals. As the clinical usefulness of rarer IFA patterns is established, these antigens may be included in future EIA methods [2]. One of these antigens might be the dense fine speckles 70 antigen (DFS70), which is associated with a dense fine speckled pattern in IFA that has promising discriminatory properties in systemic autoimmune diseases [25]. However, we did not observe any patients with this pattern in the present study, and this antigen was not included in our EIA method. A positive dense fine speckled pattern did not receive a separate classification, and positive sera may have been classified as simple speckled.

Weak positive reactions are less likely to yield concordant results between IFA and EIA techniques, as illustrated in Table 4. A two-step serial titration of positive IFA results allows for some degree of quantization. However, EIA directly provides quantitative results, simplifying the interpretation of clinically doubtful borderline reactions.

Newer methods, such as suspension arrays, simultaneously allow for multiplexing and the direct individual quantification of numerous antigens. Several companies have developed these assays, and their overall results correlate well with each other [19, 26–29]. A comparison between a multiplex assay with nine antigens and ELISA revealed 99% and 94.7% agreement in a cohort of 37 Sjögren's syndrome patients and 96 healthy controls, respectively [19]. However, a study comparing IFA against a fully automated multiplex assay with 13 antigens [29] revealed a discrepancy that was likely due to the choice of antigens and absence of standardization. The authors reported a kappa coefficient agreement of 0.31 when using IFA to assess an unselected hospital cohort of 1,004 patients; in comparison, an anti-dsDNA EIA yielded a kappa coefficient of agreement of 0.66.

## 5. Conclusions

In conclusion, quantitative EIA-based ANA techniques perform as well as (or as poorly as) IFA-based ANA techniques. The EIA methods appear to have limitations identifying nuclear and some speckled ANA reactivity, whereas the IFA techniques exhibit a limited detection of antibodies against dsDNA and SSA/Ro. The two methods are not equivalent, and both will likely produce false-negative or false-positive reactions in some cases. However, it is not clear whether these differences are clinically relevant. Quantitative automated systems for ANA screening could be used for primary screening. When more relevant antigens are identified, evaluated, and subsequently included into new EIA techniques, the use of classical IFA may diminish further.

## Figures and Tables

**Table 1:** Clinical diagnoses and results of EIA and IFA. The combined results of anti-dsDNA, ANA screening, and the seven individual antigens are referred to as “EIA-combined.” The IFA classification was based on the results from the primary laboratory and (if performed) from the secondary laboratory.

Diagnosis	dsDNA EIA			ANA screen EIA			EIA-combined			IFA		
	NEG	EQV	POS	NEG	EQV	POS	NEG	EQV	POS	NEG	EQV	POS
ANA-associated disease (*n* = 44, 18%)	38	2	4	26	2	16	28	1	15	28	2	14
SLE (*n* = 8)	5		3	4	1	3	4		4	4		4
Juvenile RA (*n* = 8)	8			7	1		8			6	1	1
Raynaud (*n* = 5)	5			2		3	3		2	4		
Vasculitis (*n* = 5)	5			4		1	5			5		
Autoimmune hepatitis (*n* = 4)	3	1		2		2	2		2	3	1	
Dermatomyositis/polymyositis (*n* = 3)	3			3			3			3		
Scleroderma (*n* = 3)	3			1		2	1		2	1		2
UCTD (*n* = 3)	2	1		2		1	1	1	1			3
MCTD (*n* = 3)	2		1			3			3			3
Discoid lupus (*n* = 2)	2			1		1	1		1	2		

Non-ANA-associated disease (*n* = 157, 46%)	150	2	5	149	5	3	149	2	7	137	12	8
Rheumatoid arthritis (*n* = 48)	46		2	44	2	2	44		4	36	9	3
Back pain (*n* = 36)	34		2	34	2		34		2	35	1	
Arthritis (*n* = 34)	32	1	1	32	1	1	32	1	1	29	1	4
Osteoarthrosis (*n* = 13)	13			13			13			13		
Polymyalgia rheumatica (*n* = 11)	10	1		11			10	1		10	1	
Arthralgia/myalgia (*n* = 7)	7			7			7			7		
Ankylosing spondylitis (*n* = 5)	5			5			5			4		1
Giant cell arthritis (*n* = 3)	3			3			3			3		

Other diseases (*n* = 185, 36%)	177	2	6	168	8	9	166	5	14	169	8	8
Neurological disorder (*n* = 58)	53			56	2		52	1	5	56	2	
Miscellaneous (*n* = 50)	50			44	3	3	46	1	3	43	4	3
Malignancy (*n* = 13)	13			12	1		12			13		
Renal disorder (*n* = 12)	11	1		12			11	1		12		
Osteoporosis (*n* = 12)	11		1	9	1	2	9	1	2	9	1	2
Inflammatory bowel disease (*n* = 10)	10			10			10			9		1
Infection (*n* = 10)	10			10			10			9		
Liver disease (*n* = 9)	8	1		8	1		8	1		9		
Vitamin D deficiency (*n* = 5)	5			1		4	2		3	3	1	1
Lung disease (*n* = 4)	4			4			4			4		
Endocrine disorder (*n* = 2)	2			2			2			2		

All (*n* = 386)	365	6	15	343	15	28	342	8	36	334	22	30

NEG: negative; EQV: equivocal; and POS: positive; MCTD: mixed connective tissue disease; RA: rheumatoid arthritis; SLE: systemic lupus erythematosus; and UCTD: undifferentiated connective tissue disease.

**Table 2:** Comparison of the immunofluorescence patterns and titers of HEp-2000 ANA testing at two independent laboratories.

Secondary laboratory	NEG	POS^?^	CEN^?^	CEN	HOM^+^	HOM	NUC^?^	NUC and HOM	NUC	SPE^?^	SPE^+^	SPE	MIT
NEG	**22**	*3 *	*1 *		*11 *	$\underline{\underline{3}}$			$\underline{\underline{2}}$		$\underline{\underline{2}}$		*1 *
CEN > 1280				**1**									
CEN > 1280 and MEM				**1**									
CEN > 1280 and SPE > 1280				**1**									
HOM^+^						**1**							
HOM > 1280						**2**							
HOM > 1280 and SPE > 1280						**1**							
HOM 1280						**1**							
HOM 160					**1**								
NUC^++^									**1**				
NUC 160	$\underline{\underline{1}}$					$\underline{\underline{1}}$			**2**				
NUC 320									**1**				
NUC 320 and SPE 160							*1 *						
NUC 640						$\underline{\underline{1}}$		**1**	**1**				
SPE 320										*1 *			
SPE												**1**	
SPE > 1280												**8**	
SPE^+++^												**1**	
MIT > 1280		*1 *											

Primary laboratory: Gentofte Hospital, and secondary laboratory: Statens Serum Institut; NEG: negative; POS: positive; CEN: centromere; HOM: homogeneous; NUC: nuclear; SPE: speckled; MIT: mitochondria; ?: uncertain reaction; +: weak reaction; ++: intermediate reaction, and +++: strong reaction. Underlining and bold font indicate agreement, italics indicate acceptable agreement, and double underlining indicates disagreement.

**Table 3:** Comparison of immunofluorescence patterns and titers between HEp-2000 immunofluorescence (Immuno Concept) and combined ANA immunoassays (Phadia). The combined EIA results from dsDNA, ANA Symphony, and the 7 subantigens (SmD, U1RNP, SSA/Ro, SSB/La, Scl-70, CENP-B, and Jo-1) are shown.

EIA-combined	NEG	POS^?^	CEN^?^	CEN	HOM^+^	HOM	NUC^?^	NUC and HOM	NUC	SPE^?^	SPE^+^	SPE	MIT
NEG	308	1			12	5	1	1	6	1	2	3	1
CENP-B				2									
dsDNA	7	1	1			2							
dsDNA	4					1							
dsDNA, SSA/Ro	1												
dsDNA, SSA/Ro	1	1											
dsDNA, La, SSA/Ro												1	
dsDNA, RnpU1, SmD, SSA/Ro						1							
SSB/La	1												
SSB/La, SSA/Ro	1											1	
SSB/La, SSA/Ro, CENP-B				1									
RnpU1	2								1			2	
RnpU1	2												
RnpU1, SSA/Ro												1	
RnpU1, SmD												1	
SSA/Ro	4	1											
SSA/Ro, CENP-B	1												
Scl-70						1							
SmD	1												

Primary laboratory: Gentofte Hospital; NEG: negative; POS: positive; CEN: centromere; HOM: homogeneous; NUC: nuclear; SPE: speckled; MIT: mitochondria; ?: uncertain reaction; +: weak reaction; ++: intermediate reaction, and +++: strong reaction. Underlining and bold font indicate agreement, italics indicate acceptable agreement, and double underlining indicates disagreement.

**Table 4:** Comparison between combined ANA immunoassay (Phadia) and HEp-2000 cell immunofluorescence (Immuno Concept). The combined results of dsDNA, ANA Symphony, and the 7 subantigens constituted the EIA-combined assay, and the combined results of IFA using HEp-2000 cells from two laboratories constituted the HEp-2000-combined assay. The results of the immunoassays were considered equivocal if the anti-dsDNA levels were between 10–15 IU/mL or if the Symphony results were between 0.7–1.0; however, the 7 subantigen results overruled those of the Symphony. The HEp-2000 results were considered equivocal if the two laboratories' results were discordant. The degree of agreement was fair (Kappa statistic = 0.29).

EIA-combined	IFA-combined			
	NEG	EQV	POS	Total
NEG	**310**	*18 *	$\underline{\underline{14}}$	342
EQV	7	**0**	*1 *	8
POS	$\underline{\underline{17}}$	*4 *	**15**	36

Total	334	22	30	388

## Abbreviations

ANA: Anti-nuclear antibodies
CENP-B: Centromere protein B
CI: Confidence interval
DFS70: Dense fine speckles 70 antigen
dsDNA: Double-stranded DNA
EIA: Enzyme immunoassay
ELISA: Enzyme-linked immunosorbent assay
HEp-2: Human epidermoid cancer cells
IFA: Indirect immunofluorescence assay
Jo-1: Histidyl-t-RNA synthetase
MCTD: Mixed connective tissue disease
RNP70 A, C: Small nuclear ribonucleoprotein complexes, 70 kDa, A, C polypeptides
Scl-70: DNA topoisomerase I
SLE: Systemic lupus erythematosus
SmD: Smith's antigen (a family of RNA-binding proteins)
SSA/Ro: Sjögren's Syndrome A antigen/small ribonucleoprotein particle (Ro 52 and 60 kDa)
SSB/La: Sjögren's Syndrome B antigen/Lupus antigen, La ribonucleoprotein domain family, member 3
U1RNP: U1 nuclear ribonucleoprotein (mixture of recombinant RNP70, A, C)
UCTD: Undifferentiated connective tissue disease
RA: Rheumatoid arthritis.
